# Relationship of Quantitative Measures of Jumping Performance with Gross Motor Development in Typically Developed Preschool Children

**DOI:** 10.3390/ijerph19031661

**Published:** 2022-01-31

**Authors:** Jing-Ling Wang, Shih-Hen Sun, Hsiu-Chen Lin

**Affiliations:** 1 Department of Physical Therapy, Lin Shin Medical Corporation Lin Shin Hospital, Taichung 408346, Taiwan; u9942851@cmu.edu.tw; 2 Department of Special Education, National Taichung University of Education, Taichung 403454, Taiwan; shsun@mail.ntcu.edu.tw; 3 Department of Physical Therapy, Graduate Institute of Rehabilitation Science, China Medical University, Taichung 406040, Taiwan

**Keywords:** preschool children, gross motor development, motion analysis, jumping performance

## Abstract

Jumping is a key movement developing in the preschool period, but limited studies have reported the determinants of jumping performance and its relationship with gross motor development. This study aimed to determine the correlations among jumping performance, quantitative parameters of jumping, and gross motor development in preschool children. Twenty-one preschool children were recruited from one kindergarten, and fifteen of them with complete data were further analyzed. The quantitative parameters of standing long jump (SLJ) and standing vertical jump (SVJ) were measured using a video-based motion capture system. The gross motor development was measured using the Preschooler Gross Motor Quality Scale (PGMQ). The Spearman’s rho value and a linear regression model were used to determine the relationships among the jumping performance, the quantitative measures, and the total PGMQ scores. The results indicate that the jumping performances were significantly correlated with the takeoff velocity, which was predicted by trunk inclination before takeoff in SLJ and by the ranges of trunk inclination during jumping in SVJ. Regression analysis showed that the preschool children with higher normalized jump height had better gross motor development, and that the jump performance and the gross motor development were directly or indirectly predicted by the slope of the hip-to-ankle angle plot during pre-takeoff. In conclusion, this study identifies key components of jumping in jumping performance and gross motor development in preschool children for physical education.

## 1. Introduction

Preschool is a crucial period for young children to develop many fundamental movements and to learn and gradually master various motor skills. Several studies have indicated the importance of motor skills in physical activity, and this relationship could present early in children [1,2,3]. Motor skills are categorized by different aspects, such as muscle groups and developmental taxonomies. In terms of developmental taxonomies, motor skills can be categorized into non-locomotor stability, locomotor, and manipulative skills [4]. Non-locomotor stability is the ability to maintain or control axial movements, which is the basis for locomotor and manipulative skills. There is a consensus that children automatically acquire locomotor skills. However, a mature level of locomotor skills is hard to achieve without practice, encouragement, or proper instructions. Lacking a mastered level of specific locomotor skills will impede further development of these skills [5]. Manipulative skills such as ball skills involve a delivery of force between a person and an object. Both locomotor and manipulative skills are essential in life. The development of locomotion and manipulation competencies will be the foundation for participation in various sports activities such as running, jumping, or ball exercises.

Typically developed preschool children possess the abilities of walking, running, jumping, and hopping, and these performances are commonly tested during this period [6]. Jumping is a diverse and functional locomotion skill with two basic types: the standing vertical jump (SVJ) and the horizontal standing long jump (SLJ). SVJ is widely applied in sports, while SLJ is commonly used as one of the fitness tests for leg muscle strength [6]. However, SVJ without arm swing is suggested to be more reliable for measuring leg muscle strength in preschool children [7]. Both SVJ and SLJ are usually considered as crucial activities in the motor development of preschool children. In addition, jumping skill has also been reported to be related to the activity level of school children [2,8].

The major difference between SVJ and SLJ is the direction of projection. SVJ projects mainly in the upward direction, while SLJ projects in both the forward and upward directions [9]. Previous studies have discussed the biomechanical determinants of SVJ and SLJ in school children and adults, but seldom in preschool children. In school children, jumping performances in SVJ were related to anthropometric characteristics [10,11] and leg power [12]. In contrast, the jumping distance of SLJ in school children was related to sex, age, body mass index, and the takeoff distance and takeoff speed of the body’s center of mass (COM). In addition, the maximal shoulder extension angle and shoulder joint angle at takeoff were correlated with jumping distance [13]. Because of the low skill proficiency in SLJ, school children showed a high fail rate in extending the arms forcefully forward and upwards, reaching full extension above the head, with the arms thrust downwards on landing [14]. In adults, the jumping performance in SVJ was related to a proximal-to-distal strategy [15], arm-swing, an increase in lower extremity work [16], and leg power [17], as with school children. Additionally, jumping performances in adults were also related to countermovement [16,18] as well as trunk inclination [18,19], presenting as a mature jumping pattern. Previous studies also found SLJ performance in adults to be related to anthropometric characteristics [10], takeoff angle [20], arm-swing [21,22], and general muscle strength [23]. An investigation on variability, i.e., levels of skillfulness, in the application of force showed similarities in children and adults during SVJ without arms; however, greater variability was observed in children during countermovement jumps (SVJ with arms) [24]. Additionally, the kinematic parameters related to proficiency in SLJ varied between preschool children and adolescents [25]. These results suggest that the level of maturation of participants and task complexity may also influence the jumping performance.

As well as using biomechanical determinants, the development of jumping skills can also be evaluated using qualitative scales. Two qualitative scales of gross motor development in preschool children have been reported in recent years: the Preschooler Gross Motor Quality Scale (PGMQ) [26] and the Children’s Activity and Movement in Preschool Study (CHAMPS) Motor Skill Protocol [27]. Both scales established content validity through literature reviews and expert panels. CHAMPS used the Test of Gross Motor Development, 2nd edition (TGMD-2) as the prototype to evaluate children aged from 3 to 5 years old. CHAMPS only includes two categories—locomotor and manipulative skills. PGMQ, on the other hand, covers a wider range of preschool ages and categories: children aged 3 to 6 years old and three functional categories of developmental taxonomies including balance, locomotion, and object manipulation subscales. PGMQ’s categories comprise various items (four in balance, eight in locomotion, and five in object manipulation), and there are four to six scoring criteria for each item. The total score for the PGMQ scale is 84. Previous studies showed that the PGMQ scale demonstrated satisfactory internal consistency, inter-rater reliability and intra-rater reliability, construct validity, and concurrent validity with the Peabody Developmental Motor Scales, Second Edition [26] and TGMD-2 [28].

Both quantitative and qualitative measures have been reported to be references for jumping performance and general development. Few studies have reported quantitative descriptions of the developing and controlling processes for jumping skills during the preschool period [29,30]. Most previous studies focused on the relationships between the jumping performance and quantitative variables in school children or adults. A few studies have reported the correlations between quantitative and qualitative performances in later childhood [31,32], but not in preschool children. Therefore, this current study aimed to determine the correlations among the jumping performance, the quantitative parameters of jumping, and gross motor development using the PGMQ scale in preschool children.

## 2. Materials and Methods

### 2.1. Participants

Typically developed children aged 4 to 6 years old were recruited from a kindergarten in Taichung, Taiwan, using convenience sampling. The Preschool Child Development Checklist screening test developed by Taipei City Government was used to exclude children with developmental delay problems. Children with any neuromuscular or musculoskeletal problems that may have affected their motor performances were also excluded. The purpose and experimental procedures of this study were explained to the teachers and parents, and informed consent was obtained before the data collection started. This study was approved by the Institutional Review Board of the China Medical University Hospital (DMR99-IRB-335-1).

### 2.2. Quantitative Data Collection and Analysis

The quantitative analysis of the jumping performances was obtained using a video-based motion capture system (MaxTRAQ, Innovision Systems, Inc., Columbiaville, MI, USA) with four Basler monochrome cameras at four corners. The child wore athletics clothes but had bare feet. Twenty-seven reflective markers were first attached to specific bony landmarks, including the midpoint of the posterior superior iliac spine, both sides of the anterior superior iliac spine, the midpoint of the lateral thigh, the femoral lateral epicondyle, the femoral medial condyle, the tibial tuberosity, the lateral malleolus, the medial malleolus, the heel, the 2nd metatarsal head, the 5th metatarsal head, the acromion process of the scapula, the humeral lateral epicondyle, and the dorsal midpoint between the ulnar styloid process and the radial styloid process. The jumping task was demonstrated by a researcher first, and then the child repeated the jump three times. The integrity of the collected data was confirmed, and then the data were included for further data processing. The video data from each camera were first digitized in the MaxTRAQ 2D environment, and then reconstructed in MaxTRAQ 3D to obtain the movement trajectories of the bony landmarks in space. Afterwards, the coordinate system of each segment was defined and used to calculate several quantitative measures using the MATLAB program (MathWorks, Inc., Natick, MA, USA). The calculated variables included the jump distance, jump height, takeoff velocity, takeoff angle, trunk inclination, and slope of the angle–angle plots for the lower extremities before takeoff. The jump distance and jump height were considered the outcome measures of the jumping performance in SLJ and SVJ, respectively. They were calculated using the recorded trajectories of the body’s center of mass (COM), represented by the center of the pelvis [33,34], and then normalized by the subject’s leg length. The movement of the body’s COM at the moment of takeoff was also used to calculate the takeoff velocity (TOV) and takeoff angle (TOA). The trunk movements were frequently considered in relation to the jumping performance, especially the inclination range in the anteroposterior direction. Therefore, the maximum trunk forward inclination before takeoff (TkBTO), the maximum trunk backward inclination during flight (TkDF), and the range (TkROM) between the above two angles were measured. At the moment of touchdown, the positions of the body’s COM and the foot were used to calculate the touchdown angle (TDA); the inclination angle from the horizontal of the thigh segments was also calculated as the dominant (dTHA) and non-dominant thigh angle (ndTHA). The dominant leg was determined by kicking a ball.

**Figure 1:**
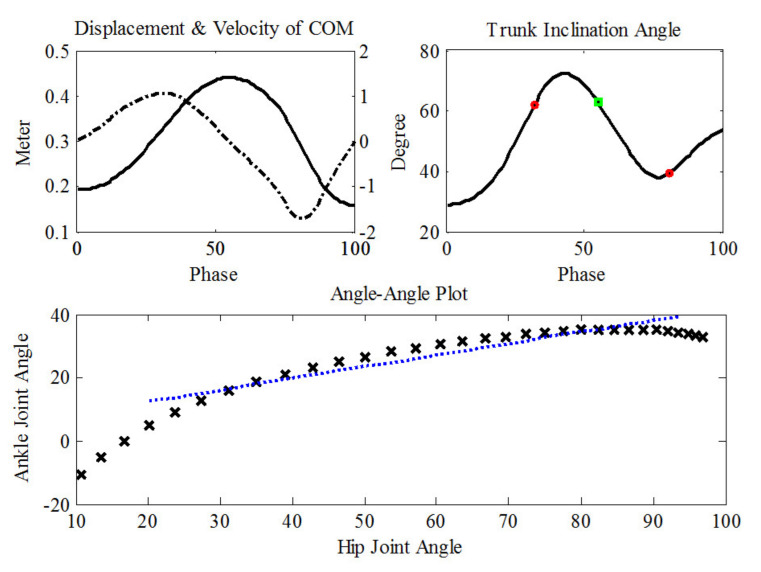
Upper panel: the peaks of the velocity of estimated COM (dash-dotted line) were used to define the moment of takeoff and landing (red circle), and the displacement (solid line) was used to define the COM’s highest point (green square). Lower panel: the hip-to-ankle angle–angle plot (black crosses) and linear regression (blue dotted line). The slope of the regression line was used to investigate the inter-joint coordination during the pre-takeoff phase.

The angle–angle plot is the angular time series of two joints [35], and it portrays the coordination between two joints by plotting one versus the other [36]. The slope of the angle–angle plot during a period indicated the average contributions of the two joints. In this study, we analyzed the period from the lowest point of the body’s COM before takeoff to the moment of takeoff, in order to explicate the coordination between joints in the lower extremities. Then, the calculated variables for analysis included the slope of the hip-to-knee joint plot (HK), the slope of the hip-to-ankle joint plot (HA), and the slope of the knee-to-ankle joint plot (KA) (Figure 1). The slope of the angle–angle plot was calculated for the dominant leg (dHK, dHA, dKA), the non-dominant leg (ndHK, ndHA, ndKA), and the mean of both legs.

### 2.3. Gross Motor Development Assessment

The total PGMQ score (PGMQ-Total) was used to examine the general level of gross motor development [26], although horizontal jumping was included as one of the testing items in the locomotion subscale of PGMQ but vertical jumping was not. The maximum score available in the PGMQ is 84, with 41 in the locomotion subscale, 25 in the object manipulation subscale, and 18 in the balance subscale. A higher score indicated a better motor skill quality. All participants were scored by two well-trained physical therapists independently on the same day during the quantitative assessment.

### 2.4. Statistical Analysis

The correlations between jump height/distance, all the quantitative variables, and the total PGMQ score were analyzed using nonparametric correlation analysis in SPSS software, to obtain the Spearman’s rho. For those variables with significant correlations, linear regression analysis was used to further establish the relationships among the jump performance, the quantitative measures, and the total PGMQ score. The stepwise method was selected to control the sequences of the variable entering the regression equation. The statistical significance was set at 0.05 in this study.

## 3. Results

Twenty-one preschool children (7 boys and 14 girls) aged 4 to 6 years were enrolled in this study, but six of them were excluded due to incomplete data. Therefore, fifteen children (4 boys and 11 girls) were included in the analysis. Their basic anthropometric characteristics, jump performances, and qualitative scores are summarized in Table 1.

The nonparametric correlations of the jump performance and PGMQ-Total score with the quantitative variables in SLJ are shown in Table 2. The children with longer jump distance had a higher TOV and larger TkROM, a smaller TOA, a smaller dTHA, a smaller THA, a smaller TKBTO, and a smaller SKA in SLJ. The children with higher total scores of PGMQ had a smaller ndHA, a smaller HA, and a smaller ndKA in SLJ.

The nonparametric correlations of the jump performance and PGMQ-Total score with the quantitative variables in SVJ are shown in Table 3. The results indicate that the children with a higher jump height had a higher zTOV, a higher TOV, a larger TkROM, a smaller TkBTO, a smaller dHA, a smaller HA, and a smaller dKA in SVJ. The children with higher total scores of PGMQ had a higher jump height, a higher zTOV, a higher TOV, a larger TkROM, a smaller TkBTO, a smaller dHA, a smaller HA, a smaller dKA, and a smaller KA in SVJ.

The stepwise regression results for SLJ and SVJ are shown in Table 4 and Table 5. The results demonstrate that TOV predicts jump performance, i.e., jump height and jump distance. TOV is predictable from TkBTO as well as ndKA in SLJ and from TkROM in SVJ. The total PGMQ scores are predictable from ndHA in SLJ and from TkROM in SVJ.

## 4. Discussion

This study aimed to determine the correlations among jumping performance, the quantitative parameters of jumping, and gross motor development in preschool children. The results of this study show that TOV had a strong correlation with jumping performance in preschool children. Other quantitative variables, including TkBTO, TOA, TkROM, THA, dTHA, and KA in SLJ, as well as dHA, dKA, TkROM, TkBTO, and HA in SVJ, were also correlated with jumping performance. The gross motor development, evaluated using PGMQ, was correlated strongly with TkBTO, TkROM, TOV, and dHA, and moderately with dHA, KA, HA, and jump height in SVJ. Nevertheless, the total PGMQ score was only correlated strongly with ndHA and HA, and moderately with ndKA in SLJ.

The mean jump distance before normalization with leg length was 0.91 m in our 4 to 6-year-old preschool children. The performance matched the mean jump distance of 0.91 m in the middle class of kindergarten in Liu’s study on preschool boys in Taiwan [37], and also fell within the reported range in three other studies showing a jump distance of 0.69 m in 3 to 6-year-old children in Andalusia [38], 0.81 m in 5 to 6-year-old children [9], and 1.08 m and 0.97 m in 6.5-year-old boys and girls in the Republic of Croatia [39]. The mean jump height before normalization with leg length was 0.17 m in this study. This was similar to the findings in the study by Zhao et al. [40], but smaller than the mean jump height of 0.25 m in the 6-year-old children in the Taiwan group in Wang’s study [41], since our children were younger.

### 4.1. The Determinants of Jumping Performance

**Figure 2:**
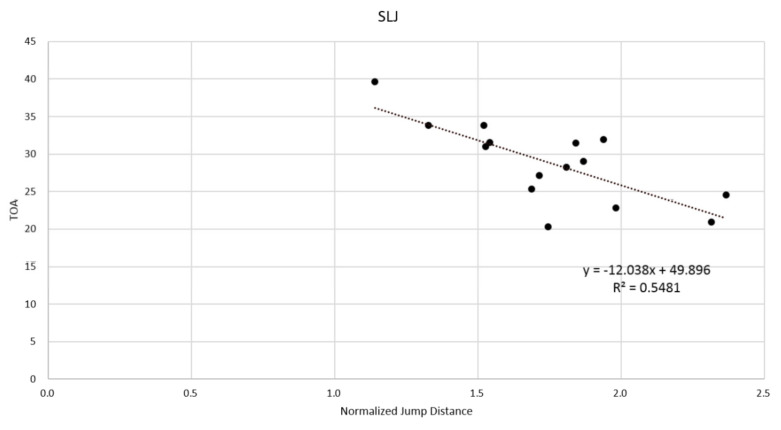
The relationship between the takeoff angle (TOA) and the normalized jump distance (NJD) in standing long jump (SLJ).

In this study, the TOV, TOA (assessed only in SLJ), TkBTO and TkROM were correlated with jump performance. According to the principles of projectile motion, the horizontal distance of jumping can be maximized when takeoff angle is 45 degrees. However, Wakai and Linthorne suggested that the calculated optimum takeoff angle was 19–27 degrees in their study. This is because takeoff speed declines with an enhanced takeoff angle for overcoming one’s body weight [20]. Consistent with the study of Wakai and Linthorne, our results show that a smaller takeoff angle of around 20–25° resulted in a better jump distance (Figure 2). Therefore, the TOV and TOA could be key determinants of jumping performance.

Several studies have showed the involvement of trunk movement during jumping activities. The study of Vanrenterghem et al. [19] showed that performing a vertical jump with restricted trunk inclination in athletic male adults significantly changed the joint angular displacement and the joint power in the lower extremities, and decreased the jump height, which suggests the crucial role of trunk movements. Lees et al. indicated that a vertical jump with arm swing increases the trunk inclination and thus the jump height in athletic adult males [42]. The study of Kopper et al. [18] also showed that the trunk position in a vertical jump with a small knee flexion angle altered the proximal-to-distal activation pattern, while a countermovement jump retained the proximal-to-distal activation pattern and thus enhanced jump height. The study of Zhao et al. [40] also found that the hip muscle played an important role during a vertical jump in preschool children. The study of Fukashiro et al. compared the trunk flexion angles between SLJ and SVJ in male football players (age: 24.9 ± 2.4 years), indicating a larger trunk flexion angle before takeoff and a larger trunk angular displacement in a horizontal jump than in a vertical jump [43]. In our study, similar trunk parameters (e.g., TkBTO and TkROM) were found to be the predictors of the key determinant, TOV, in SLJ and SVJ. Specifically, the TkBTO was predictable from TkROM and TOA in SLJ, which indicated that the trunk position before takeoff is a determinant factor for the TOV. In contrast, the range of trunk movement is a determinant factor for the TOV in SVJ, which indicates that the trunk movement could provide the power for projection. The results of this study confirm the importance of trunk movements for jumping in preschool children.

In this study, the movement pattern of the dominant leg before takeoff in SVJ showed moderate associations with jump performance and gross motor development. A previous study investigated the landing kinematics of single-leg hopping between the dominant and non-dominant leg, showing that hip extension in the dominant leg was smaller than in the non-dominant leg, but the dominant leg had a longer hopping distance [44]. In this study, both the dHA and dKA in SVJ correlated with the jump height, which indicated that a coordinated movement pattern between joints in the dominant leg would facilitate the motor performance, and that this might related to the bi-articular muscle control and also the strength feature of the dominant leg. Lanshammar and Ribom investigated muscle strength between the dominant and non-dominant leg, and indicated that the dominant leg favored leg extension and the non-dominant leg favored leg flexion [45]. Leg extension provides the progression force of jumping, which could be the reason that only the movement pattern of the dominant leg was related to jumping performance. Additionally, jump performance in SLJ was correlated with KA, but in SVJ it was correlated with HA. This might indicate that different joint coordination patterns before takeoff in SLJ and SVJ appeared in the preschool period.

### 4.2. The Relationship between Quantitative Parameters of Jumping and Gross Motor Development in Preschool Children

The relationships between the quantitative variables of jumping and the gross motor development in preschool children have not previously been extensively investigated in the literature. The results of the present study demonstrate moderate correlations between the total PGMQ score and jumping performance only in SVJ, which indicates that SVJ would be more appropriate to differentiate gross motor development in preschool children. Jumping performance in SLJ did not have a significant correlation with the total PGMQ score, which might be due to the complex techniques in SLJ, making it hard to achieve a specific level of SLJ in the preschool period.

In this study, correlations between the PGMQ-Total score and quantitative parameters of jumping in SLJ were found only in the specific slopes of angle–angle plots, including ndHA, HA, and ndKA. A previous study investigated the landing movement of a drop jump, showing a larger dynamic knee valgus angle in the dominant leg than in the non-dominant leg during unilateral landing, and showing more stability in the non-dominant leg [46]. Another study discussed the landing movement in unilateral and bilateral drop jumps and showed a greater flexion angle as well as smaller flexion excursion of the hip and knee joint in the non-dominant leg than in the dominant leg during bilateral landing. [47]. Both studies investigated the landing phase of a drop jump and showed the stability feature of the non-dominant leg. The results of this current study indicate that the movement pattern before takeoff in the non-dominant leg when performing SLJ correlates with the gross motor development. This might suggest that the coordination and stability provided by the non-dominant leg could assist the preschool child to achieve the completion of every motor component while performing SLJ.

Correlations between the PGMQ-Total score and quantitative parameters in SVJ were found in TOV, trunk movement, including TkBTO and TkROM, and the specific slopes of angle–angle plots, including dHA, HA, dKA, and KA. The gross motor development displayed the same features as the jump performance regarding the correlations with the trunk movement during SVJ in preschoolers. Both HA and KA in SVJ were negatively correlated with the PGMQ-Total score, which indicated that, with a higher gross motor level, preschoolers use more movements at the hip and knee joints rather than at the ankle joint during the pushing phase before takeoff. In addition, HA in SLJ was also negatively correlated with the PGMQ-Total score, indicating the importance of the hip joint movement control in gross motor development. This agreed with the study of Mana et al., which also identified the hip flexion angle before takeoff as a predictor for jumping distance in preschoolers. Although the hip flexion angle was defined by the trunk segment and thigh segment in Mana’s study, the results still showed the importance of the trunk and hip movement in SLJ [29]. Zhao et al. investigated biomechanical variables of a vertical jump in preschool children and showed that the hip and knee were the main contributors before takeoff [40]. The movement patterns of the hip and knee relative to the ankle joint before takeoff are two key elements of the jumping performance in SVJ and correlate with the gross motor development. Specifically, the contribution from the hip joint movements might be more important in the jumping performance than that from the ankle joint during the preschool period.

### 4.3. Limitations in This Study

A major limitation of this study was the use of convenience sampling to recruit our participants and the small sample size. Though the gender distribution of these children was uneven, the PGMQ-Total score demonstrated no significant differences during normal development [48]. Additionally, compared with similar groups in previous studies, the basic anthropometric characteristics (body height and body weight) and jump performances were in accordance with those of preschool children generally. A second limitation is that we did not collect kinetic data, due to the setup in the kindergarten. However, the contribution of different joints of the lower limb was instead illustrated using the coordination between joints in the angle–angle plots. Lastly, the age range for preschool children is usually defined as between 3 and 6 years. However, we did not include 3-year-old children because most of them cannot complete these two jumping tasks correctly, indicating that jumping ability is developed gradually between 3 and 4 years of age.

## 5. Conclusions

This study found that several quantitative parameters were correlated with the performance in two types of jumping, including TOV, TkBTO, and TkROM. Most of these parameters were found to be correlated with the gross motor development only in SVJ. Regression analysis further showed that the preschool children with higher normalized jump height had better gross motor development. Regarding the movement pattern, the regression analysis showed that the jump performance and the gross motor development could be directly or indirectly predicted from the slope of the hip-to-ankle angle plot during pre-takeoff.

### Practical Application

Despite the small sample size, the results of this study indicate that SVJ is an appropriate activity to include in physical education in the preschool period because the practice of SVJ might assist gross motor development. When instructing preschoolers to perform SVJ, the emphasis should be placed on hip movement before takeoff as well as the range of trunk motion from inclination to extension. In contrast, when instructing them to perform SLJ, the focus should be placed on knee movement before takeoff as well as trunk inclination before takeoff, in order to enhance the performance. This study has established the relationships between jumping performances, quantitative variables, and gross motor development. In future studies, a similar analysis could also be used for children with developmental delay, to further understand their motor abilities.

## Figures and Tables

**Table 1:** The basic anthropometric characteristics, jump performances, and qualitative scores (mean ± standard deviation) of the preschoolers.

Characteristics		Mean ± Standard Deviation
Anthropometric characteristics	Age (years)	5.65 ± 0.65
	Body height (m)	1.08 ± 0.07
	Body weight (kg)	20.73 ± 3.64
Standing long jump	Jump distance (m)	0.91 ± 0.18
	Normalized distance (% leg length)	175.4 ± 32.9
	Takeoff velocity, TOV (m/s)	2.46 ± 0.29
	Takeoff angle, TOA (deg)	28.78 ± 5.35
Standing vertical jump	Jump height (m)	0.17 ± 0.04
	Normalized height (% leg-length)	33.4 ± 7.8
	Takeoff velocity, TOV (m/s)	1.83 ± 0.28
PGMQ qualitative score ^1^	Locomotion subscale score	35.87 ± 2.33 (range: 31–40)
	Object manipulation subscale score	19.33 ± 3.85 (range: 11–24)
	Balance subscale score	17.07 ± 1.39 (range: 13–18)
	PGMQ-Total score	72.27 ± 5.26 (range: 65–81)

^1^ PGMQ = Preschooler Gross Motor Quality Scale.

**Table 2:** Spearman’s correlation in standing long jump (SLJ).

Variables	NJD		PGMQ-Total	
	Spearman’s Rho	*p*-Value	Spearman’s Rho	*p*-Value
Normalized jump distance, NJD	1.000		0.327	0.235
Takeoff velocity, TOV	0.917 **	0.000	0.299	0.279
Takeoff angle, TOA	−0.657 **	0.008	−0.390	0.151
Touchdown angle, TDA	0.496	0.060	0.338	0.219
Thigh angle at touchdown (dominant leg), dTHA	−0.600 *	0.018	−0.226	0.418
Thigh angle at touchdown (non-dominant leg), ndTHA	−0.357	0.191	0.083	0.770
Mean value of both thigh angles at touchdown, THA	−0.618 *	0.014	−0.007	0.98
Trunk inclination before takeoff, TkBTO	−0.757 **	0.001	−0.284	0.306
Trunk inclination during flight, TkDF	−0.236	0.398	0.083	0.770
Range of motion of trunk inclination, TkROM	0.636 *	0.011	0.214	0.445
Slope of hip-to-knee joint plot (dominant leg), dHK	0.075	0.791	−0.391	0.149
Slope of hip-to-knee joint plot (non-dominant leg), ndHK	0.025	0.930	−0.327	0.235
Mean value of slope of hip-to-knee joint plot of both legs, HK	0.122	0.666	−0.323	0.240
Slope of hip-to-ankle joint plot (dominant leg), dHA	−0.174	0.536	−0.46	0.084
Slope of hip-to-ankle joint plot (non-dominant leg), ndHA	−0.386	0.155	−0.705 **	0.003
Mean value of slope of hip-to-ankle joint plot of both legs, HA	−0.437	0.103	−0.600 *	0.018
Slope of knee-to-ankle joint plot (dominant leg), dKA	−0.374	0.170	−0.227	0.417
Slope of knee-to-ankle joint plot (non-dominant leg), ndKA	−0.496	0.060	−0.534 *	0.040
Mean value of slope of knee-to-ankle joint plot of both legs, KA	−0.525 *	0.044	−0.483	0.068

* Correlation is significant at the 0.05 level (2-tailed). ** Correlation is significant at the 0.01 level (2-tailed).

**Table 3:** Spearman’s correlation in standing vertical jump (SVJ).

Variables	NJH		PGMQ-Total	
	Spearman’s Rho	*p*-Value	Spearman’s Rho	*p*-Value
Normalized jump height, NJH	1.000		0.516 *	0.049
Takeoff velocity in vertical direction, zTOV	0.890 **	0.000	0.576 *	0.024
Takeoff velocity, TOV	0.872 **	0.000	0.650 **	0.009
Trunk inclination before takeoff, TkBTO	−0.554 *	0.032	−0.697 **	0.004
Trunk inclination during flight, TkDF	−0.239	0.390	−0.368	0.177
Range of motion of trunk inclination, TkROM	0.568 *	0.027	0.668 **	0.007
Slope of hip-to-knee joint plot (dominant leg), dHK	−0.302	0.274	−0.316	0.251
Slope of hip-to-knee joint plot (non-dominant leg), ndHK	−0.204	0.466	−0.210	0.452
Mean value of slope of hip-to-knee joint plot of both legs, HK	−0.247	0.376	−0.246	0.377
Slope of hip-to-ankle joint plot (dominant leg), dHA	−0.614 *	0.015	−0.605 *	0.017
Slope of hip-to-ankle joint plot (non-dominant leg), ndHA	−0.304	0.270	−0.313	0.257
Mean value of slope of hip-to-ankle joint plot of both legs, HA	−0.518 *	0.048	−0.517 *	0.049
Slope of knee-to-ankle joint plot (dominant leg), dKA	−0.602 *	0.017	−0.551 *	0.033
Slope of knee-to-ankle joint plot (non-dominant leg), ndKA	−0.419	0.120	−0.332	0.227
Mean value of slope of knee-to-ankle joint plot of both legs, KA	−0.479	0.071	−0.524 *	0.045

* Correlation is significant at the 0.05 level (2-tailed). ** Correlation is significant at the 0.01 level (2-tailed).

**Table 4:** Stepwise regression in standing long jump (SLJ).

Tested Variables	Predictor	R Square	Adjusted R Square	F	Sig.	Unstandardized Coefficients		Standardized Coefficients	Sig.
Model						B	Std. Error	Beta	
NJD, normalized jump distance									
1	TOV	0.841	0.829	69.009	0.000	1.053	0.127	0.917	0.000
TOV, takeoff velocity									
1	TkBTO	0.522	0.486	14.22	0.002	−0.019	0.005	−0.723	0.002
2	TkBTO	0.723	0.677	15.666	0.000	−0.02	0.004	−0.74	0.000
	ndKA					−0.165	0.056	−0.448	0.012
TkBTO, trunk inclination before takeoff									
1	TkROM	0.516	0.478	13.838	0.003	−0.732	0.197	−0.718	0.003
2	TkROM	0.727	0.681	15.966	0.000	−0.574	0.162	−0.563	0.004
	TOA *					0.97	0.319	0.485	0.010
TkROM, range of motion of trunk inclination									
1	HA	0.355	0.305	7.156	0.019	−27.544	10.296	−0.596	0.019
PGMQ-Total									
1	ndHA	0.399	0.353	8.645	0.011	−11.299	3.843	−0.632	0.011
ndHA, slope of hip-to-ankle joint plot (non-dominant leg)									
1	HA	0.854	0.843	76.063	0.000	1.198	0.137	0.924	0.000
2	HA	0.999	0.999	10,477.4	0.000	1.976	0.017	1.524	0.000
	dHA					−0.994	0.018	−0.711	0.000

* TOA = takeoff angle, dHK = slope of hip-to-knee joint plot of dominant leg, ndHK = slope of hip-to-knee joint plot of non-dominant leg, HA = mean value of slope of hip-to-ankle joint plot of both legs.

**Table 5:** Stepwise regression in standing vertical jump (SVJ).

Tested Variables	Predictor	R Square	Adjusted R Square	F	Sig.	Unstandardized Coefficients		Standardized Coefficients	Sig.
Model						B	Std. Error	Beta	
NJH, normalized jump height									
1	zTOV	0.831	0.818	64.066	0	0.274	0.034	0.912	0.000
zTOV, takeoff velocity in vertical direction									
1	TOV	0.938	0.933	196.25	0	0.892	0.064	0.968	0.000
2	TOV	0.957	0.949	132.173	0	1.037	0.084	1.125	0.000
	TkROM					−0.004	0.002	−0.208	0.042
TOV, takeoff velocity									
1	TkROM	0.569	0.536	17.144	0.001	0.017	0.004	0.754	0.001
TkROM, range of motion of trunk inclination									
1	TkBTO	0.637	0.61	22.861	0	−0.666	0.139	−0.798	0.000
TkBTO, trunk inclination before takeoff									
1	dHA *	0.69	0.667	29.002	0	33.122	6.15	0.831	0.000
PGMQ-Total									
1	TkROM	0.55	0.515	15.868	0.002	0.306	0.077	0.741	0.002
TkROM, range of motion of trunk inclination									
1	TkBTO	0.637	0.61	22.861	0	−0.666	0.139	−0.798	0.000
2	TkBTO	0.743	0.7	17.339	0	−0.448	0.157	−0.536	0.015
	TOV					19.012	8.57	0.417	0.047
TkBTO, trunk inclination before takeoff									
1	dHA *	0.69	0.667	29.002	0	33.122	6.15	0.831	0.000

* dHA = slope of hip-to-ankle joint plot (dominant leg).
